# 

**Published:** 1843-10-14

**Authors:** 


CONTENTS.

M. Rostan's Clinical Lecture, - - 229
A case of Retained Placenta, - - 230
Case of Ascites. By R. M. Denig, M.D. 231
*Bibliographical Notices.*
Special Anatomy and Histology. By W. E. Horner, M. D., &c. - - 232
Practical Manual of the Diseases of the Heart and the Great Vessels. By F. A. Aran, Interne of the First Class of the Hotel Dieu, &c. &c. Translated from the French. By William A. Harris, M. D., - - - - - - - 232
The Hospitals and Surgeons of Paris. By F. Campbell Stewart, M. D. - 233
*Retrospect of the Medical Sciences.*
New Operation for artificial Pupil. By J. B. Estlin, - - - - - 234
Statistics of Puerperal Convulsions, - 234
New Astringent Principle of Rhatany, - 234
Mr. Estlin on Injuries of the Iris, - 235
External Application of Iodine in Croup, 236
The Importance of Abstinence from Bread in Diabetis Mellitus. By Theophilus Thompson, M. D. - - - - 236
Dr. James on the Relief of Deformities produced by Cicatrices in the Neck after Burns, - - - - - - 236
Pilula Ferri Composita, - - - 237
Meeting of Medical Gentlemen in Suffolk, 237
Venous Inflammation of the Uterus, - 238
Dr. Gibson on the Means of Determining the Relative Position of the Thoracic and Abdominal Vicera, - - 238
Rupture of the Uterus—Recovery, - 239
Dr. Ricord on the Treatment of Gonnorrhœal Ophthalmia, - - - 239
New System of Hæmospasy, - - 240
Formula for Rheumatism, - - - 240
Sugar in the Urine of Gouty and Dyspeptic Persons, - - - - - 240

